# Involvement of FoxQ1 in NSCLC through regulating EMT and increasing chemosensitivity

**DOI:** 10.18632/oncotarget.2103

**Published:** 2014-06-13

**Authors:** Jian Feng, Liqin Xu, Songshi Ni, Jun Gu, Huijun Zhu, Haiying Wang, Shu Zhang, Wei Zhang, Jianfei Huang

**Affiliations:** ^1^ Department of Respiratory Medicine, Affiliated Hospital of Nantong University. Nantong, Jiangsu, China; ^2^ Department of Pathology, Affiliated Hospital of Nantong University. Nantong, Jiangsu, China

**Keywords:** Non-small cell lung cancer, FoxQ1, Epithelial-mesenchymal Transition, Chemosensitivity, Apoptosis

## Abstract

Forkhead box Q1 (FoxQ1) is a member of the forkhead transcription factor family. High expression of FoxQ1 has been associated with several cancers including non-small cell lung cancer (NSCLC), but its role in the development of NSCLC is not clear. In this study, we investigated the effect of FoxQ1 up-regulated and down-regulated *in vitro* and *in vivo*, and the role of FoxQ1 in regulating epithelial-mesenchymal transition (EMT) in NSCLC, providing evidence that FoxQ1 could be a potential therapeutic target in NSCLC. NSCLC cells with silenced FoxQ1 had decreased cell proliferation, migration and invasion in cell culture and delayed growth of xenograft tumors in mice compared with corresponding control cells. The NSCLC cells downregulated for FoxQ1 induced the expression of apoptosis-associated proteins and reduction of anti-apoptotic protein expression. Downregulation of FoxQ1 promoted the expression of epithelial markers and decreased several mesenchymal markers *in vitro* and *in vivo*. In addition, FoxQ1 was associated with resistance to conventional chemotherapeutic agents. In contrast, FoxQ1 overexpressed elicited converse effects on these phenotypes *in vitro* and *in vivo*. Our findings define a key role for FoxQ1 in regulating EMT and increasing chemosensitivity in NSCLC.

## INTRODUCTION

Lung cancer is the leading cause of cancer-related mortality worldwide. Patients who receive surgically complete resection and several regimens of chemotherapy have been documented to show improved survival. However, cancer metastasis and resistance to treatment (including radiotherapy, chemotherapy and targeted therapy) remain the two major causes of the poor survival of lung cancer patients [1]. The prognosis of these patients is ominous, with 5-year survival rates of 10% [2].

Development of lung cancer involves multiple genetic and epigenetic changes that lead to transformation of normal cells into cancer cells. FoxQ1, a member of the forkhead transcription factor family [3-5], is a well-characterized candidate oncogene located on chromosome 6p23-25 [3] that plays an important role in the etiology of human cancer [4, 6-8], especially in lung cancer [9]. Several recent studies [6-8, 10] demonstrated the correlation between increased FoxQ1 expression with poor prognosis for many human cancers, including breast cancer, hepatocellular carcinoma and colon cancer. FoxQ1 was shown to regulate EMT and function in breast cancer [6]. Suppression of FoxQ1 in benzyl isothiocyanate-mediated inhibited EMT in human breast cancer cells [11]. Another study reported that FoxQ1 promotes hepatocellular carcinoma metastasis through provoking EMT by transactivating ZEB2 and VersicanV1 expression [7]. These studies showed that FoxQ1-mediated EMT functions in and promotes cancer metastasis. We have shown that FoxQ1 was upregulated in NSCLC compared with peritumoral tissues. The expression of FoxQ1 in adenocarcinoma was higher than in squamous cell carcinoma, and FoxQ1 overexpression influenced poor prognosis in NSCLC and was associated with EMT [9].

Substantial evidence suggests that EMT plays a prominent role in chronic diseases, such as organ fibrosis and cancer [12, 13]. EMT is characterized by upregulation of mesenchymal markers such as fibronectin [14, 15], vimentin (VIM) [14, 16-18] and S100 calcium-binding protein A4 (S100A4) [19, 20], and downregulation of E-cadherin [16, 21, 22] and Mucin 1 (MUC1) [18, 23]. EMT has been noted as a crucial event in tumor metastasis and invasion in epithelial-derived cancers [21, 24-26], including NSCLC [27-30]. It is a dynamic process underlying metastasis through promoting acquisition of migratory and invasive abilities [31, 32]. These findings have provided a connection between EMT, apoptosis, and drug resistance.

Here we explored the effect of FoxQ1 silencing and overexpression *in vitro* and *in vivo*, and the corresponding changes in EMT. Biochemical and transcriptomic investigations allowed us to identify the molecular pathways involved in the FoxQ1-driven phenotype.

## RESULTS

### Association of FOXQ1 Expression with NSCLC

**Figure 1:**
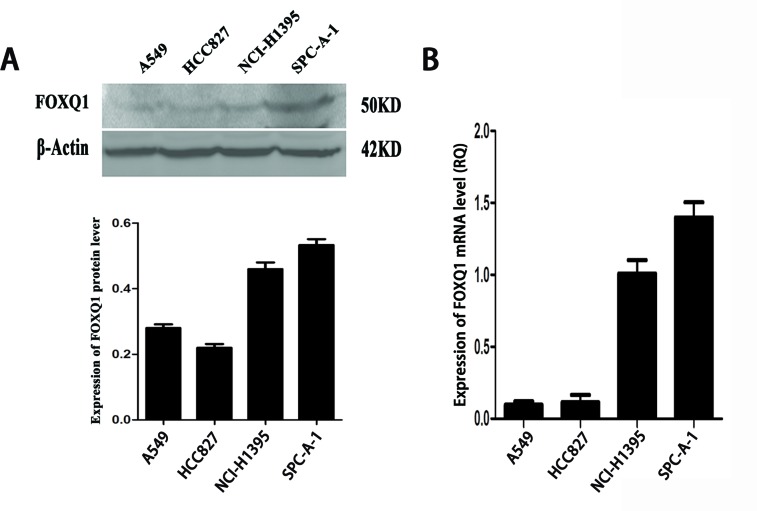
Expression of FoxQ1 protein and mRNA in four non-small cell lung cancer cell lines A). FoxQ1 protein expression in four cell lines. β-actin was used as loading control. B). FoxQ1 mRNA expression in four cell lines normalized to GAPDH.

Our previous research showed upregulation of FoxQ1 expression in NSCLC tissues and association with EMT [9]. Here we examined FoxQ1 mRNA and protein expression in four NSCLC lines (SPC-A-1, NCI-H1395, HCC827, and A549). Quantitative RT-PCR (qRT-PCR) and western blot results showed higher expression of FoxQ1 in SPC-A-1 and NCI-H1395 cell lines than HCC827 and A549 cell lines (Figure 1).

### Effect of Depletion or Enforcing FoxQ1 Expression on Cell Proliferation, Migration and Invasiveness of Lung Carcinoma Cells

**Figure 2:**
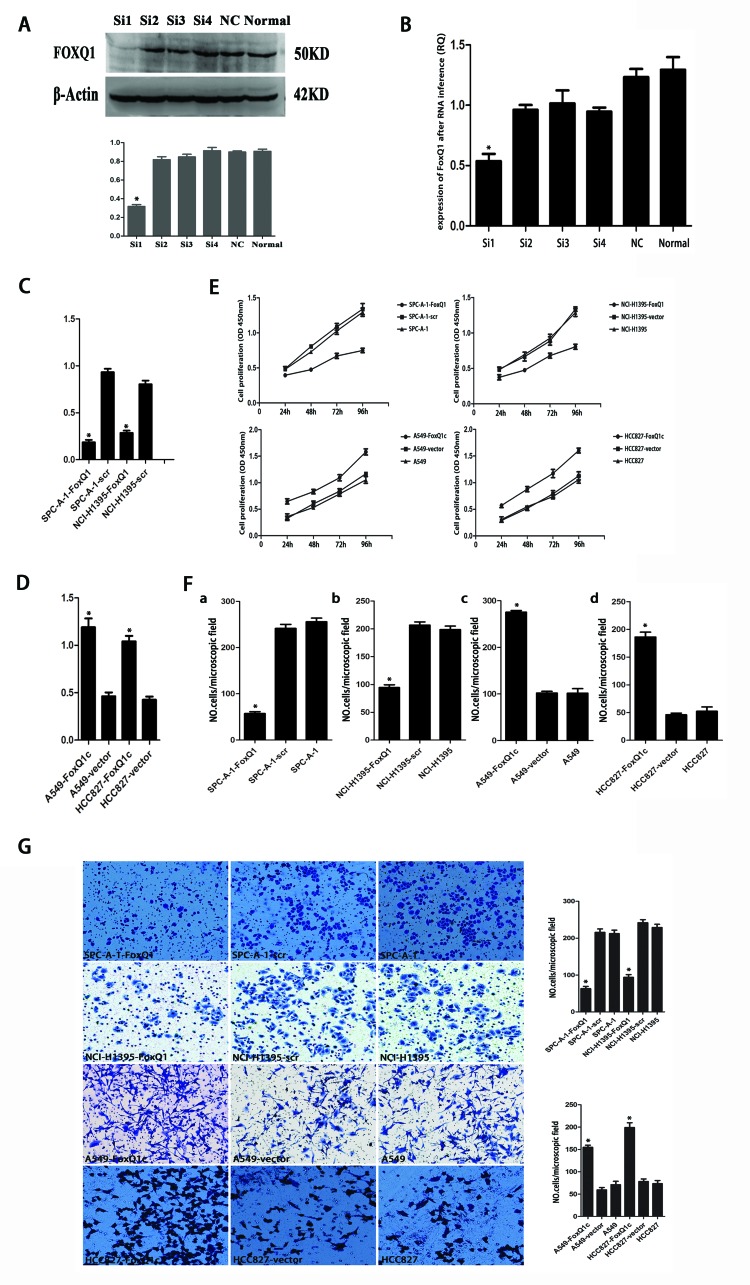
Effect of depleting or enforcing the expression of FoxQ1 on cell proliferation, migration and invasiveness of lung carcinoma cells A) qRT-PCR and B) western blot were used to select the most effective silencing siRNA targeting human FOXQ1. C) Two stably transfected cell lines silenced for FoxQ1 were established by G418 screening. G418-resistant clones were examined by qRT-PCR. D) Two stably transfected cell lines overexpressing FoxQ1 were identified by G418 screening, and qRT-PCR was used to confirm G418-resistant clones. E) The proliferation ability of the four experimental cell lines was examined using CCK-8 at 450 nm. Specifically, 5 × 10^3^ cells were seeded in 100 μL of medium per well into 96-well plates (three wells per each group). Then, 10 μL of CCK8 solution was added to the culture medium in each well after 24h, 48h, 72h and 96h. Then cells were incubated for 3 h another. The absorbance was determined at 450 nm wavelength. F) Migration and G) invasion ability were presented as total number of cells that migrated to the bottom chamber without or with the transwell-precoated matrigel, respectively, as calculated in at least six random fields (total magnification ×200) per filter. (*P<0.05).

We next explored the functional consequence of altering the expression of FoxQ1 in NSCLC cell lines by examining four different sequences of siRNA targeting human FoxQ1 and negative control siRNA. qRT-PCR and western blot identified FoxQ1#1 as the most potent sequence for silencing (Figure 2, A and B). We transfected the PGPH1/GFP/Neo vector carrying FoxQ1#1 siRNA into SPC-A-1 and NCI-H1395 cell lines, which had shown high FoxQ1 protein expression, and used G418 screening to establish the two stable cell lines SPC-A-1-FoxQ1 and NCI-H1395-FoxQ1. Corresponding control cells were generated with vector with scrambled siRNA. We also introduced a FoxQ1 cDNA expression vector into HCC827 and A549 cell lines, which had shown low FoxQ1 protein expression, and screened with G418 to generate cells stably overexpressing FoxQ1 protein, the A549-FoxQ1c and HCC827-FoxQ1c cell lines. The G418-resistant clones were examined by qRT-PCR, and decreased FoxQ1 mRNA expression was confirmed with FoxQ1 shRNA expression and increased levels confirmed upon FoxQ1 cDNA expression (Figure 2, C and D).

Next we evaluated cell proliferation, migration and invasion of the four experimental cell lines. Cell lines silenced with FoxQ1 shRNA (SPC-A-1-FoxQ1 and NCI-H1395-FoxQ1) had lower proliferative abilities than the corresponding controls and normal controls. However, cells that overexpressed FoxQ1 protein (A549-FoxQ1c and HCC827-FoxQ1c) had higher levels of cell proliferation than the corresponding vector control and normal control cells (Figure 2E). In addition, proliferation was inhibited in SPC-A-1-FoxQ1 and NCI-H1395-FoxQ1 cells after 48 h and 72 h, and proliferation was elevated in A549-FoxQ1c and HCC827-FoxQ1c cell lines at 24 h, compared with the control cells.

In the migration and invasion assays, more A549-FoxQ1c and HCC827-FoxQ1c cells migrated through the membrane in the migration chamber with or without the Transwell-precoated Matrigel than A549-vector and HCC827-vector cells. However, fewer SPC-A-1-FoxQ1 and NCI-H1395-FoxQ1 cells migrated compared to SPC-A-1-scr and NCI-H1395-scr cells (Figure 2, F and G), with statistical significance (*P* < 0.05). These results indicated that silencing FoxQ1 expression decreased cell proliferation, migration and invasion of NSCLC cells, while overexpression of FoxQ1 increased these biological behaviors.

### Constitutive Activation and Silencing of FoxQ1 on Growth of NSCLC in Nude Mice

**Figure 3:**
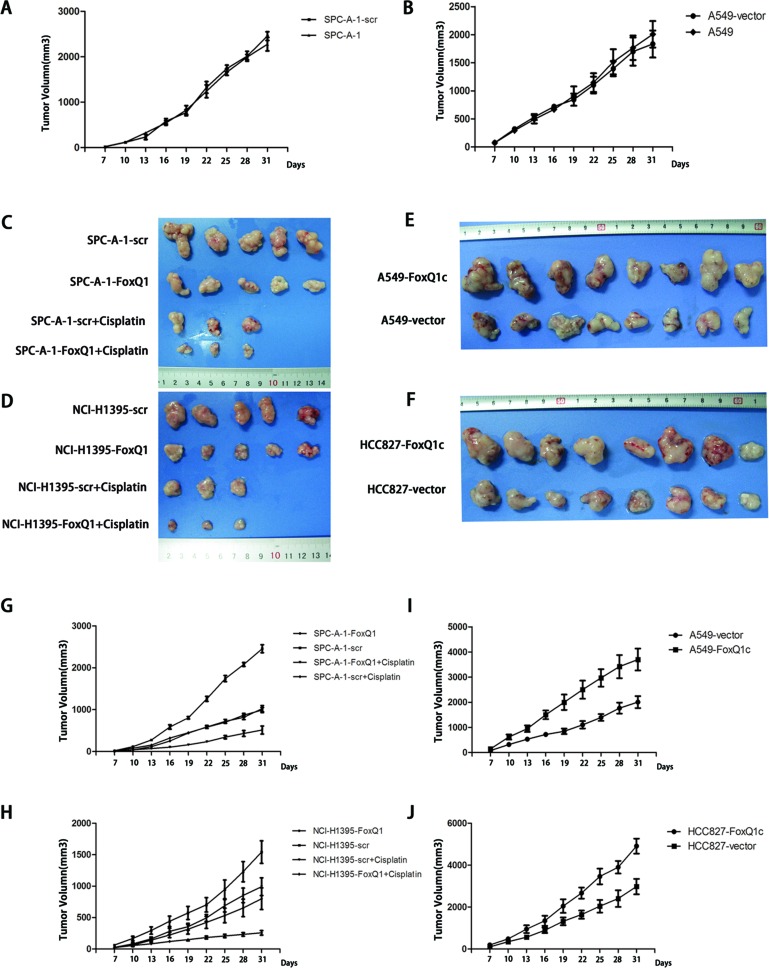
Constitutive activation and silencing of FoxQ1 on growth of NSCLC in nude mice Experimental cells were subcutaneously injected into BALB/c nude mice. A–B) The tumor growth curves of the negative control group and normal control group (*P* > 0.05, A: *P*=0.369, B: *P*=0.634). C–F) Images of tumors in FoxQ1 silenced groups, FoxQ1 overexpressing groups, and corresponding controls groups, including intraperitoneal injection of cisplatin groups. G–H) Growth of tumors produced by subcutaneous injection of mice with SPC-A-1-FoxQ1 (G, *P*=0.044) or NCI-H1395-FoxQ1 (H, *P*=0.041) cells, and vector control cells, as well as cisplatin treatment groups. The tumors from mice that received cisplatin and stable transfection cell injection were smaller than those either treated with cisplatin or transfected alone (*P*=0.015 and *P*=0.037 in Figure 3G and Figure 3H respectively). Error bars = 95% confidence interval, CI. I-J) The tumor growth curves of HCC827-FoxQ1c (I, *P*=0.027) and A549-FoxQ1c (J, *P*=0.020) human NSCLC tumors in nude mice compared with control tumors.

To investigate the effect of FoxQ1 on tumor growth, xenografts were established by subcutaneously injecting different cell lines into BALB/c athymic nude mice. As shown in Figure 3A and B, we first examined tumor growth in negative control and normal control groups, excluding the cytotoxicity of dsRNA and nonspecific RNAi mechanism in the transfection process, and no significant difference in tumor growth curve was detected at each point in time (*P* > 0.05, A: *P*=0.369, B: *P*=0.634). Cells in which the expression of FoxQ1 was silenced (SPC-A-1-FoxQ1 and NCI-H1395-FoxQ1), their corresponding control cells (SPC-A-1-scr and NCI-H1395-scr) and normal control cells were subcutaneously injected into the bilateral flank, and tumor size was measured and recorded every three days. Tumor formation was detected in all eight mice per cell line (Figure 3C and D). Growth curves showed that the tumor volume in SPC-A-1-FoxQ1 and NCI-H1395-FoxQ1 cells silenced for FoxQ1 expression was much lower than that in corresponding control and normal control mice. The average tumor volume per mouse injected with SPC-A-1-FoxQ1 cells was 689 mm^3^ at 28 days compared with 2438 ± 188 mm^3^ tumor volume formed by SPC-A-1-scr cells (Figure 3G, *P*=0.044). The tumor volume in NCI-H1395-FoxQ1 group mice was reduced (423 ± 110 mm^3^) compared with tumors formed by NCI-H1395-scr (1879 ± 188 mm^3^) (Figure 3H, *P*=0.041). Additionally, the tumors from mice that received cisplatin and stable transfection cell injection were smaller than those either treated with cisplatin or transfected alone (*P*=0.015 and *P*=0.037 in Figure 3G and Figure 3H respectively). Interestingly, we found that tumor growth speed was noticeably accelerated in SPC-A-1-scr, NCI-H1395-scr, SPC-A-1, and NCI-H1395 groups at certain periods (21 d in SPC-A-1 cell line and 27 d in NCI-H1395 cell line).

In addition, overexpression of FoxQ1 protein increased the growth of HCC827-FoxQ1c and A549-FoxQ1c tumors in nude mice compared with control tumors (Figure 3, E and F). The average tumor volume per mouse reached 2856 mm^3^ at 35 days in the A549-FoxQ1c group, while 1022 ± 88 mm^3^ tumor sizes were observed with A549-vector cells (Figure 3I, *P*=0.027). Similar trends were observed with the xenograft implantation of HCC827-FoxQ1c cells (Figure 3J, *P*=0.020). It is worth noting that six of sixteen mice in the overexpression groups showed involvement of ribs, and this might be attributed to enhanced invasion as a result of increased FoxQ1 expression.

### FoxQ1 Expression and Apoptosis *In Vitro* and *In Vivo*

**Figure 4:**
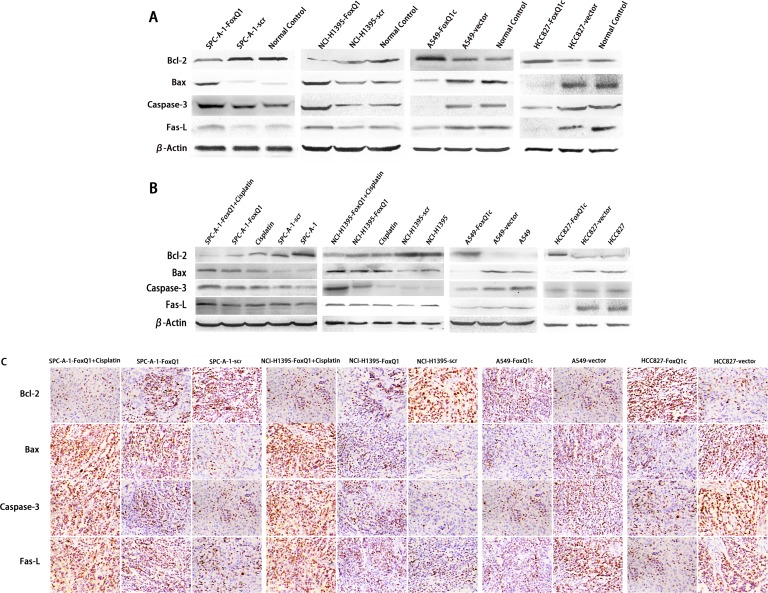
FoxQ1 expression and apoptosis in vitro and in vivo (A) Western blot analysis of apoptosis-associated protein expression in FoxQ1 silenced or overexpressed cells and tissues. β-actin was used as a loading control. (B) Western blot of apoptosis-associated protein expression in frozen tissues, including upon cisplatin combination treatment. β-actin was used as a loading control. (C) Analysis of apoptosis-associated protein expression on TMA was performed by immunohistochemistry staining (magnification ×200). The result was consistent with (A) and (B).

To explore the underlying mechanism by which FoxQ1 induces lung tumor growth, apoptosis-associated protein expression was analyzed in FoxQ1 silenced or overexpressed cells. Expressions of the pro-apoptotic protein Bax, Caspase-3 and Fas-L were increased at different degrees in SPC-A-1-FoxQ1 and NCI-H1395-FoxQ1 cells, whereas expression of anti-apoptotic protein Bcl-2 was decreased, compared with corresponding control cells. Additionally, Bax, Caspase-3 and Fas-L expressions were reduced in A549-FoxQ1c and HCC827-FoxQ1c cells, while Bcl-2 protein was elevated compared with corresponding control cells (Figure 4A).

We also investigated apoptosis-associated protein expression *in vivo*. Western blot results of frozen tissues were consistent with the above results (Figure 4B). Bax, Caspase-3 and Fas-L were increased and Bcl-2 was decreased in SPC-A-1-FoxQ1 and NCI-H1395-FoxQ1 groups, whereas the opposite results were observed in A549-FoxQ1c and HCC827-FoxQ1c groups, compared with corresponding control groups. Cisplatin was administered by intraperitoneal injection to three of eight mice from FoxQ1 silenced groups. Tissues from cisplatin-treated tumors had more apoptosis than normal controls, and when shRNA and cisplatin was combined, apoptosis levels were higher (Figure 4B).

For immunohistochemistry staining, Bax, Caspase-3, Fas-L, Bcl-2 were tested on TMA. Various levels of protein staining in the nuclei were observed. Higher Bax, Caspase-3, Fas-L expression and lower Bcl-2 expression were observed in SPC-A-1-FoxQ1 and NCI-H1395-FoxQ1 groups, and apoptotic cell amounts in shRNA and cisplatin combined tissues were highest (Figure 4C). In the A549-FoxQ1c and HCC827-FoxQ1c groups, Bax, Caspase-3, Fas-L expression decreased and Bcl-2 expression increased, compared with corresponding control groups (Figure 4C).

Together our results suggest that silencing FoxQ1 expression might promote apoptosis in non-small lung cells, and the combination of cisplatin and targeting FoxQ1 could be more effective in promoting apoptosis and should be explored in future studies.

### FoxQ1 Promotes EMT *In Vitro* and *In Vivo*

**Figure 5:**
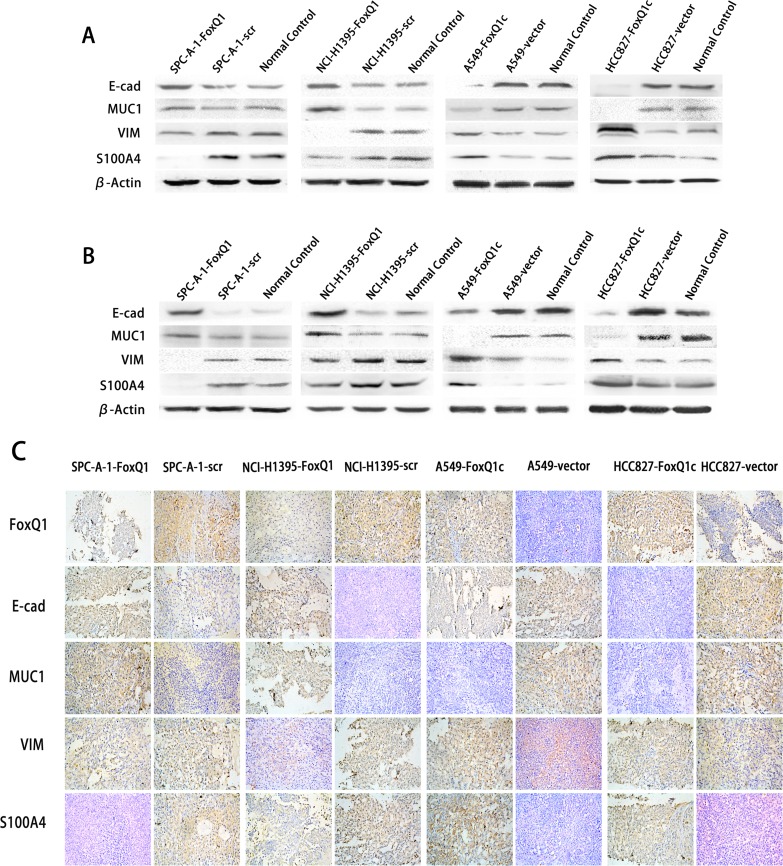
FoxQ1 promotes EMT in vitro and in vivo A) Western blot analysis of protein expression of two epithelial indicators and two mesenchymal markers in four cell lines silenced or overexpressed for FoxQ1. β-actin was used as the loading control. B) Western blot for the expression of EMT indicators in frozen tissues. C) Immunohistochemistry staining on TMA for FoxQ1 and four EMT marker proteins (magnification ×200).

EMT is involved in the invasive ability of transformed epithelial cells [35] and our previous study showed that high FoxQ1 expression correlated with loss of E-cadherin expression and anomalous positivity of VIM and S100A4 [9]. Compared with SPC-A-1-scr and NCI-H1395-scr control cells, stable silencing of FoxQ1 in SPC-A-1 and NCI-H1395 cells significantly increased expression of CDH1/E-cadherin protein, and MUC1 was also increased in NCI-H1395-FoxQ1 cells, with no difference in SPC-A-1-FoxQ1 cells (Figure 5A). Changes in VIM and S100A4 were also observed in both cell lines upon FOxQ1 silencing. However, overexpression cell lines A549-FoxQ1c and HCC827-FoxQ1c showed reduced E-cadherin and MUC1 expression, and increased levels of mesenchymal proteins. These data show for the first time that in NSCLC, FoxQ1 can induce downregulation of E-cadherin expression and upregulation of VIM and S100A4 expression at endogenous levels.

We further investigated FoxQ1-induced EMT *in vivo*. FoxQ1 and the four EMT indicators were analyzed by western blot in frozen tissues and IHC staining on TMA upon different treatments (Figure 5, B and C). As shown in Figure 5C, FoxQ1 was poorly expressed in SPC-A-1-FoxQ1 and NCI-H1395-FoxQ1 groups, and showed nuclear and cytoplasm localization. E-cadherin and MUC1 were increased, with expression in the cell membrane and a combination of the plasmalemma and cytoplasm, respectively, of NSCLC cells. Moreover, SPC-A-1-FoxQ1 and NCI-H1395-FoxQ1 groups also displayed decreased mesenchymal markers VIM and S100A4, which were partly localized in the cytoplasm, and a combination of the nucleus and cytoplasm. In A549-FoxQ1c and HCC827-FoxQ1c groups, we found the opposite results (Figure 5C). Similar data were obtained in western blot results from tumor tissues (Figure 5C).

Together these data indicate that FoxQ1 promotes EMT in NSCLC, and this role in promoting EMT might be another important function for FoxQ1 in tumorigenesis.

### FoxQ1 Overexpression Confers Resistance to Chemotherapy-Induced Apoptosis

**Figure 6:**
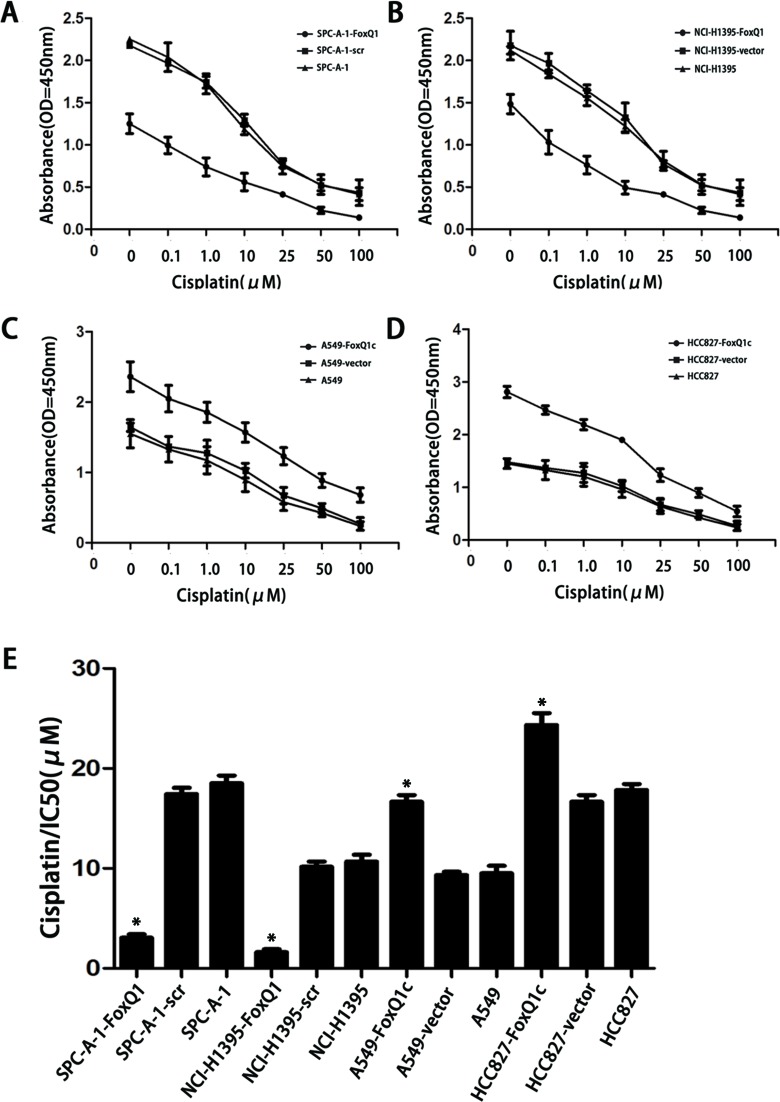
FoxQ1 overexpression confers resistance to chemotherapy-induced apoptosis A–B) FoxQ1 depletion in SPC-A-1-FoxQ1 and NCI-H1395-FoxQ1 cells contributed to increased apoptotic response to cisplatin. (Error bars = 95% confidence interval, CI; *P*=0.003 and *P*=0.007 respectively). C–D) FoxQ1 overexpression in A549-FoxQ1c and HCC827-FoxQ1c cells was correlated with increased resistance to cisplatin. (Error bars = 95% CI; *P*=0.001 and *P*=0.000 respectively). E) Half-maximal inhibitory concentration (IC50) values of cisplatin. IC50 values were determined by use of CCK-8, as described above. (Error bars = 95% CI; *P<0.05).

Cancer cells undergoing EMT have recently been connected to chemoresistance [36]. We investigated the association between FoxQ1 expression and chemoresistance to four chemotherapeutic agents in NSCLC cells and explored whether FoxQ1 plays a role in drug-induced apoptosis. FoxQ1 depletion in SPC-A-1-FoxQ1 and NCI-H1395-FoxQ1 cells contributed to increased apoptotic response to various levels of chemotherapeutic reagents commonly used in lung cancer, including GEM, DDP, DOX, and PEM (Figure 6, A: *P*=0.003 and B: *P*=0.007, part data not shown). Conversely, FoxQ1 overexpression correlated with increased resistance the drugs (Figure 6, C: *P*=0.001 and D: *P*=0.000). Specifically, the IC50 was higher in A549-FoxQ1c and HCC827-FoxQ1c cells than control cells. However, the IC50 in SPC-A-1-FoxQ1 and NCI-H1395-FoxQ1 cells was lower than in control cells (Figure 6E *P*<0.05). Hence, downregulation of FoxQ1 might increase the sensitivity of NSCLC cells to chemotherapeutic reagents, and may be related to our above result demonstrating that decreasing FoxQ1 expression could induce tumor cell apoptosis.

## DISCUSSION

Several studies have demonstrated the importance of EMT and decreased E-cadherin in NSCLC, but the specific contributions of FoxQ1 to the progression of this disease have not been fully explored. In our previous research, we showed FoxQ1 overexpression in NSCLC with poor prognosis, and its association with EMT [9].

Here we confirmed significant upregulation of FoxQ1 mRNA and protein in NSCLC tissues and found that high expression was associated with poor prognosis. This correlation is consistent with the consequences of FoxQ1 overexpression described in our *in vitro* and *in vivo* results, including E-cadherin downregulation previously shown to be related with poor prognosis in NSCLC [37]. Our *in vitro* and *in vivo* findings shed light on how FoxQ1 promotes tumor progression in NSCLC. Higher expression of FoxQ1 in adenocarcinoma than squamous cell carcinoma was verified [9], and four adenocarcinoma cell lines were used to model the potential proliferative role of FoxQ1 silencing or overexpression in NSCLC. The functional effects of FoxQ1 knockdown in two high expression cell lines were consistent: decreased proliferation, migration and matrigel invasion, and decreased growth of xenograft NSCLC tumors in nude mice. The specificity of these responses are in contrast to results from the two FoxQ1 overexpression cell lines. This is consistent with recent studies reporting FoxQ1 overexpression in breast cancer [4, 6] and colorectal cancer [6, 10], and that high expression of FoxQ1 enhanced tumorigenicity and tumor growth [10]. However, these findings are in contrast to the results of Kaneda et al. [10], who showed that decreased FoxQ1 expression in H1299 cells increased proliferation by downregulated p21^Cip1/Waf1^ expression. This implies that the FoxQ1 target gene specificity is context specific [38].

EMT is a critical event in tumor invasion and metastasis in epithelial-derived cancers [21, 24-26]. During oncogenesis, epithelial tumor cells undergo EMT and display enhanced migratory capacity and invasiveness [35, 39]. Our study described another important finding: that FoxQ1 promotes EMT in independent models of human NSCLC cells and nude mice. Our results demonstrated that FoxQ1 expression was significantly associated with EMT in lung cancer cells as well as the TMA of tumor models. FoxQ1 repression led to changes of epithelial cell morphology and increased cellular size. This was paralleled by cytoskeleton rearrangements as well as increased expression of several junction proteins. Whether the cytoskeletal changes and alterations in cellular junctions are primary or associated with the morphological alterations remain to be resolved. When we silenced FoxQ1, E-cadherin expression increased and VIM and S100A4 mesenchymal markers decreased. E-cadherin is a critical switch in EMT [6] and regulates cell shape and cellular size [40]. Functional loss of E-cadherin is a hallmark of EMT [12, 41]. MUC1 expression was inconsistent in two stably silenced lines, with no differences in the TMA. Additionally, overexpression of FoxQ1 could downregulate E-cadherin and MUC1 levels, and upregulate VIM and S100A4 levels. These results are consistent with the report of a variable relationship between FoxQ1 and E-cadherin levels in other malignancies [6]. This phenomenon is likely associated with the tumor microenvironment and may reflect interactions with other transcription repressors. E-cadherin is mainly inactivated by transcriptional repression at the promoter level through several transcription factors, included the snail and zeb family. Moreover, the Forkhead transcription factors have been shown to be involved in regulating the plasticity of epithelial cells [42-44]. The expression and activity of these transcription factors including FoxQ1 are modulated by TGF-β signaling [29, 45, 46], which can induce EMT in many epithelial cells [47, 48]. Our research is consistent with the involvement of FoxQ1 in TGF-β1 signaling-induced EMT in NSCLC. We investigated the link between FoxQ1 and EMT at vitro cells and *in vivo* tissues levels, in keeping with our previous study on tumor TMA. Our findings describing the interplay between FoxQ1 and EMT provide significant contributions to the exploration of EMT in tumor progression and invasion.

TGF-β family members initiate and maintain EMT via activation of major signaling pathways and transcriptional regulators in extensive signaling networks in various biological systems and pathophysiological situations [49, 50]. Studies have demonstrated that genetic programs that regulate EMT control TGF-β-induced growth arrest and/or apoptosis. Once cells have adopted a mesenchymal phenotype, genes do not respond to TGF-β suppressor effects [51, 52]. Increasing evidence suggested that acquired resistance to chemotherapy is likely to correlate to EMT [53]. For instance, high E-cadherin expression and increased mesenchymal phenotype can lead to apoptosis resistance. We observed that decreased expression of FoxQ1 enhanced the sensitivity of four typical chemotherapeutic agents and inhibited tumor growth in nude mice when combined with cisplatin. Moreover, downregulated FoxQ1 expression promoted apoptosis of lung cancer cells and the apoptosis induced by cisplatin. Therefore, FoxQ1 may play a key role in multiple drug resistance in human cancer and this likely occurs via multiple mechanisms. Hence, with the connection of EMT to disease progression in cancer pathology, our study might open up a novel perspective for future cancer therapy through modulation of cellular FoxQ1 activities.

In summary, here we reveal that RNA interference against FoxQ1 inhibited proliferation, invasive and migration of NSCLC cell lines and restrained tumorigenesis and development of NSCLC. Decreasing FoxQ1 expression could induce apoptosis as occurs in NSCLC cells and mice tumors by undergoing EMT. TGF-β likely plays an important role by activating FoxQ1 to switch the response towards the induction of EMT. Additionally, FoxQ1 might be associated with resistance to conventional chemotherapy and downregulation of FoxQ1 may increase the sensitivity of chemotherapeutic reagents, with important implications in cancer progression and treatment.

This research had some limitations. FoxQ1-inference plasmids were only tested in NSCLC lines and require further validation for treatment of antibody-induced apoptosis in established animal models. In addition, tumor growth was investigated in immunodeficient mice, but whether conclusions also apply to human NSCLC patients remains to be explored. Finally, whether FoxQ1 is specific to NSCLC or plays a similar functional role in other epithelial cancers remains to be determined.

Further studies are warranted to elucidate the mechanisms involved in FoxQ1-mediated NSCLC tumorigenesis, and explore the effect of RNA/lentivirus-mediated FoxQ1-silencing on NSCLC growth in animal models and the diagnostic value of FoxQ1 protein in serum in a large cohort of NSCLC patients. Additionally, anti-FoxQ1 monoclonal antibodies could block the suppressive effect of FoxQ1 on apoptosis. Therefore, antibodies against the secreted form of FoxQ1 or its undefined receptor should be investigated for therapeutic value in NSCLC.

## MATERIALS AND METHODS

### Cell Lines and Cell Culture

Human NSCLC cell lines A549, SPC-A-1, HCC827 and NCI-H1395 were obtained from Shanghai Institute of Biochemistry and Cell Biology, Chinese Academy of Science. The four cell lines were cultured in RMPI-1640 medium (HyClone, Logan City, Utah, USA) containing 10% fetal bovine serum (FBS), 2 mM L-glutamine, 100 U/ml penicillin/streptomycin mixture (Gibco BRL, Grand Island, NY, USA) and maintained in a 5% CO_2_ humidified atmosphere at 37°C.

### Quantitative Real-Time Reverse Transcription PCR (qRT-PCR)

The methods for qRT-PCR have been previously described [33]. Total RNA was extracted from cells using Trizol reagent (Invitrogen, Carlsbad, CA, USA), and reverse transcribed to cDNA using a Revert AidTM First Strand cDNA synthesis kit (Fermentas, Glen Burnie, MD, USA) following the supplier’s instructions. The primers used for real-time RT-PCR purchased from Sangon (Shanghai, China) were as follows: FOXQ1 forward, 5′-TCGCAACTTCCATTGATT-3′ and reverse, 5′- TCACACTCAGTCATACCT-3′; GAPDH forward, 5′- TCGGAGTCAACGGATTTGGTCGT-3′ and reverse, 5′- TGCCATGGGTGGAATCATATTGGA -3′. The transcripts were quantified with SyberGreen on an ABI 7500 thermal cycler (Applied Biosystems). The PCR conditions were as follows: UDG pre-treatment at 50°C for 2 min; initial denaturation at 95°C for 10 min; and denaturation at 95°C for 15 sec, and annealing and extension at 60°C for 60 sec, for up to 40 cycles. Transcripts were normalized to GAPDH by subtracting the average GAPDH Ct values (Threshold Cycle) from the average Ct of transcripts, resulting in Ct. Target mRNA levels were determined by standard curve method and expressed as arbitrary units. The experiment was performed in triplicate.

### Western Blot

The total protein extracts from each cell line and tumor tissues were obtained using a lysis buffer (Beyotime Institute of Biotechnology, Nantong, China), and protein concentration was determined by the BSA method (Beyotime Institute of Biotechnology, Nantong, China). Equal amounts (40 μg per lane) were separated by SDS-polyacrylamide gel electrophoresis (PAGE) in 6%, 10% and 12% acrylamide gels and transferred to polyvinylidine difluoride (PVDF) membranes (Millipore Corporation, USA) at 300 mA for 2 h. The membrane was blocked in 5% fat-free milk and incubated with the following primary antibodies overnight at 4°C: rabbit anti-FoxQ1 (1:500 dilution; Abcam, UK), rabbit anti-E-cadherin (1:500; Invitrogen), monoclonal rabbit anti-EMA (1:1500; Novocastra, UK), monoclonal rabbit anti-VIM (1:2000; Invitrogen), polyclonal rabbit anti-S100A4 (1:800; Newmarker, USA), rabbit anti-Bax (1:1000 dilution; Abcam), monoclonal rabbit anti-Bcl-2 (1:500 dilution; Abcam), polyclonal rabbit anti-Caspase3 (1:1000 dilution; Abcam) and monoclonal rabbit anti-Fas (1:1000 dilution; Abcam). The secondary antibody was horseradish peroxidase-conjugated (HRP)-conjugated goat anti-rabbit antibody (1:500, Beyotime Institute of Biotechnology). After stripping, the membrane was reprobed with β-actin (1:1000, Beyotime Institute of Biotechnology) overnight at 4°C, followed by incubation with secondary antibody as above at room temperature for 2 h. Bands were visualized using an enhanced chemiluminescence system (ECL, Beyotime Institute of Biotechnology). Data were quantified by densitometry.

### SiRNA Transfection, Plasmid Constructs and Generation of Stable Cell Lines

Four different siRNA sequences targeting human FoxQ1 and negative control siRNA were designed and obtained from Shanghai Genepharma Corporation. The sequences of Si-FoxQ1 and control siRNA were as follows: FoxQ1#1 sense, 5′-GCCAAGCAAUUUCUUUAAATT-3′ and antisense, 5′-UUUAAAGAAAUUGCUUGGCTT-3′; FoxQ1#2 sense, 5′-GCAACUUCCAUUGAUUUAUTT-3′ and antisense, 5′-AUAAAUCAAUGGAAGUUGCTT-3′; FoxQ11#3 sense, 5′-GGGAACCUUUCCACACUAUTT-3′ and antisense, 5′-AUAGUGUGGAAAGGUUCCCTT-3′; FoxQ1#4 sense, 5′-CAACGGGCUACAGCUUUAUTT-3′ and antisense, 5′-AUAAAGCUGUAGCCCGUUGTT-3′. Negative control siRNA sequences were (scramble) sense, 5′-UUCUCCGAACGUGUCACGUTT-3′ and antisense, 5′-ACGUGACACGUUCGGAGAATT-3′. Cells were then transfected by Lipofectamine^TM^ 2000 (Invitrogen) according to the manufacturer’s instructions.

We selected the two most effective silencing sequences by western blot and RT-PCR analysis and ligated each sequence into the PGPH1/GFP/Neo vector. Full-length FoxQ1 cDNA was cloned into pCMV6/AC/GFP vector (OriGene, USA). Four cell lines were separately transfected with plasmids and selected by GFP sorting. Cells were then grown in complete medium containing 200 μg/ml G418 (Roche Diagnostics, Mannheim, Germany). RT-PCR was used to confirm the presence of the plasmids. Clones were isolated and expanded into cell clones after four weeks. The subcloned cells expressing Neo and FoxQ1 genes were named as SPC-A-1-FoxQ1, NCI-H1395-FoxQ1, A549-FoxQ1c and HCC827-FoxQ1c. The corresponding controls were named SPC-A-1-scr, NCI-H1395-scr, A549-vector and HCC827-vector.

### Cell Proliferation Assays

For analysis of cell proliferation, SPC-A-1-FoxQ1, NCI-H1395-FoxQ1, A549-FoxQ1c, HCC827-FoxQ1c, SPC-A-1-scr, NCI-H1395-scr, A549-vector HCC827-vector cells and normal control cells (5 × 10^3^) in 100 μL of medium were seeded per well into 96-well plates (three wells per each group). Cell proliferation was evaluated using the Cell Counting Kit-8 (CCK-8, Beyotime Institute of Biotechnology) according to the manufacturer’s instructions. Briefly, 10 μL of CCK8 solution was added to the culture medium in each well, and cells were incubated for 3 h. The absorbance was determined at 450 nm wavelength. The assays were repeated three times with triplicate samples.

### Transwell Migration and Invasion Assays

For cell invasion assays, modified Boyden Chambers consisting of Transwell-precoated Matrigel membrane filter inserts with 8 μm pores were used in 24-well tissue culture plates (BD Biosciences, Bedford, MA). Cells from different groups (1 × 10^5^) were plated onto the top of the chamber in RMPI1640 without FBS and the bottom chamber was filled with RMPI1640 containing 10% FBS as a chemoattractant. After 24 h of incubation in a 5% CO_2_ humidified chamber at 37°C, noninvading cells were removed by wiping the upper surface of the membrane with a cotton swab, and the filter membrane was fixed with 4% paraformaldehyde and stained with Exam MaSiLiang blue. The degree of invasion was quantified by counting the cells that had migrated through the membrane in at least six random fields (total magnification, ×200) per filter. Experiments were repeated three times in triplicate.

For analysis of cell migration, we use the modified Boyden Chambers without the Transwell-precoated Matrigel membrane filter, using the method performed as above.

### Tumor Formation in Nude Mice

SPC-A-1-FoxQ1, NCI-H1395-FoxQ1, A549-FoxQ1c, HCC827-FoxQ1c cells or corresponding control cells were injected subcutaneously in mice to investigate the ability to generate xenograft tumors. BALB/c athymic nude mice (4 to 6 weeks old) were purchased from Shanghai Laboratory Animal Center, China and kept in a specific pathogen-free environment. All mouse experiments followed institutional guidelines and were approved by the committee on the Ethics of Animal Experiments of Nantong University, Permit Number: SYXK (su) 2012-0030. We harvested 1 × 10^7^ cells by incubation in trypsin-EDTA, washed the cells twice with PBS, resuspended the cells in 0.2 mL of RMPI medium, and injected each cell line subcutaneously into BALB/c athymic nude mice. Eight mice were used per cell line and each mouse received two injections, each of 1 × 10^7^ cells, in the bilateral flank to form two tumors. We injected stably transfected cells into one side of each mouse and the corresponding control cells in the other side.

To explore the effect of altered FoxQ1 expression on chemotherapeutics, we selected the classical antitumor drug cisplatin. At 7 days of tumor formation, three of the eight mice in each group in which FoxQ1 was silenced were randomly selected and received an intraperitoneal injection of cisplatin (7.5 mg/kg). The remaining five mice received an injection of the same amount of saline. The tumor growth of different treatment groups was monitored until the day that mice were killed. The date at which the first grossly visible tumor appeared was recorded, and the tumor size was measured every 3 days. Two-dimensional measurements were taken with an electronic caliper after injection, and the tumor volume was calculated with the following formula: tumor volume (in mm^3^) = π/6×a × b^2^, where a is the longest diameter, and b is the shortest diameter. When a tumor reached 2.0 cm in diameter, the mouse was anesthetized by 1% pentobarbital sodium (Sigma, St. Louis, MO, USA) and photographed. The tumors were excised, weighed and measured. Half of the primary tumors were fixed in 10% formalin overnight and subjected to routine histological examination by investigators who were blinded to the tumor status. The other half was frozen at –80°C for later research.

### Tumor Tissue Microarray (TMA) and Immunohistochemistry (IHC) Staining

All tumor samples were embedded in paraffin after fixing in 10% formaldehyde for 24 h and used for constructing the TMA. A representative area of each sample was selected and 2.0 mm tissue cores were designed for constructing a TMA by Shanghai Super Biotek, China. We used hematoxylin-eosin staining (H&E) to confirm the quality of TMA sections.

IHC staining was performed as described previously [34]. Briefly, sections (4 μm) were deparaffinized and rehydrated. Antigen retrieval was performed by boiling under pressure in citrate buffer, pH 6.0, for 3 min. Non-specific binding was blocked by 5% goat serum in PBS for 15 min, and the tissues were incubated with primary antibodies as follows: rabbit anti-FoxQ1 (1:300 dilution; Abcam), rabbit anti-E-cadherin (1:120; Invitrogen), monoclonal rabbit anti-EMA (1:200; Novocastra), monoclonal rabbit anti-VIM (1:100; Invitrogen), polyclonal rabbit anti-S100A4 (1:100; Newmarker), rabbit anti-Bax (1:300 dilution; Abcam), monoclonal rabbit anti-Bcl-2 (1:250 dilution; Abcam), polyclonal rabbit anti-Caspase3 (1:300 dilution; Abcam) and monoclonal rabbit anti-Fas (1:250 dilution; Abcam). The secondary antibody was EnVision goat anti-rabbit HRP (DAKO, USA). The immunostained sections were evaluated by two trained pathologists who were unaware of our research purpose.

### Chemotherapeutic Cell Treatments

Gemcitabine (GEM), cisplatin (DDP), docetaxel (DOX), and pemetrexed (PEM) were used at 0.1–100 μM to determine the half-maximal inhibitory concentration (IC50) values in stable cell lines in which FoxQ1 was silenced or overexpressed and their corresponding controls cells. Cells (5 × 10^3^) were added to each well in a 96-well plate and cultured for 24 h. Cells were then treated with drugs for 12 h and the media was replaced by fresh medium without drugs for additional 48 h. Cell viability was measured by a Cell Counting Kit-8 (Beyotime Institute of Biotechnology) at 450 nm as above. DMSO treatment was used as a control. The survival of each cell line was compared with their corresponding control cell line. Assays were repeated three times.

### Statistical Analysis

All statistical analyses, including t-test, *X^2^*-test, and Mann-Whitney U-test, were carried out with the GraphPad Prism software (version 5; GraphPad Software, La Jolla, CA, USA) and STATA 9.0 software (Stata Corporation, College Station, TX).

### Conflict of interest statement

There are no conflicts of interest associated with this manuscript.

### Funding

This study was supported by the Technological Innovation and Demonstration of Social Undertakings Projects (HS2013021) of Nantong, China.
